# Supplementary material for: Mechanisms governing the pioneering and redistribution capabilities of the non-classical pioneer PU.1
Source: Nat Commun. 2020 Jan 21;11:402. doi: 10.1038/s41467-019-13960-2 (PMC6972792; doi:10.1038/s41467-019-13960-2)
Supplement: Supplementary file 7 — Source data [file 41467_2019_13960_MOESM7_ESM.zip › Source_Data/FigureS2/FigureS2B_MotifScanOutput/KmeansCluster3/homerResults/motif15.similar.html]

motif15

## Information for motif15

C
G
T
A
T
C
A
G
C
G
A
T
C
T
A
G
T
A
C
G
G
T
C
A
C
G
T
A
C
T
A
G
C
G
A
T
T
C
A
G
T
A
G
C
C
G
A
T
C
A
T
G
C
A
T
G
  
Reverse Opposite:  

G
T
A
C
G
T
A
C
G
C
T
A
A
T
C
G
A
G
T
C
C
G
T
A
G
A
T
C
C
G
A
T
C
A
G
T
A
T
G
C
G
A
T
C
G
C
T
A
A
G
T
C
G
C
A
T
  

|  |  |
| --- | --- |
| p-value: | 1e-67 |
| log p-value: | -1.565e+02 |
| Information Content per bp: | 1.692 |
| Number of Target Sequences with motif | 129.0 |
| Percentage of Target Sequences with motif | 2.70% |
| Number of Background Sequences with motif | 96.6 |
| Percentage of Background Sequences with motif | 0.23% |
| Average Position of motif in Targets | 111.6 +/- 51.8bp |
| Average Position of motif in Background | 100.6 +/- 49.0bp |
| Strand Bias (log2 ratio + to - strand density) | 0.0 |
| Multiplicity (# of sites on avg that occur together) | 1.00 |
| Motif File: | file (matrix) reverse opposite |

### Similar de novo motifs found

|  |  |  |  |  |  |  |  |
| --- | --- | --- | --- | --- | --- | --- | --- |
| Rank | Match Score | Redundant Motif | P-value | log P-value | % of Targets | % of Background | Motif file |
| 1 | 0.883 | T C A G C T G A C T A G C G A T C T A G A C T G T G C A C G T A G A C T A C G T T C A G A T G C C G A T | 1e-23 | -53.190000 | 0.78% | 0.05% | motif file (matrix) |
| 2 | 0.877 | C T G A C T A G C G A T C T A G T C A G C T G A C G T A A G C T C G A T T A C G A G T C A C G T | 1e-18 | -42.212689 | 0.73% | 0.07% | motif file (matrix) |
